# Metallothionein-Like Proteins and Energy Reserve Levels after Ni and Pb Exposure in the Pacific White Prawn *Penaeus vannamei*

**DOI:** 10.1155/2010/407360

**Published:** 2010-08-24

**Authors:** Gabriel Nunez-Nogueira, Catherine Mouneyrac, Alice Muntz, Laura Fernandez-Bringas

**Affiliations:** ^1^Unidad Academica Geología Marina y Ambiental, Instituto de Ciencias del Mar y Limnología, Universidad Nacional Autónoma de México, México 04510, DF, Mexico; ^2^MMS, EA2160, Faculté de Pharmacie, Université de Nantes, 1 rue G. Veil, BP 53508, 44035 Nantes Cedex 1, France; ^3^Institut de Biologie et d'Ecologie Appliquée, CEREA, Université Catholique de l'Ouest, 3 Place André Leroy, 49008 Angers, France

## Abstract

This study analyzed the changes in metallothionein-like proteins (MTLPs) and Energy Reserves (ERs) in hepatopancreas and abdominal muscle of the white prawn *Penaeus vannamei*. Realistic metal concentration exposure for 10 days to Ni and Pb in solution revealed that juvenile prawns partially induce MTLP in hepatopancreas after Pb exposure. Ni was distributed equally between soluble and insoluble fractions, while Pb was present only in the insoluble fraction, suggesting different detoxification strategy. No changes in lipids and glycogen concentration were detected under these experimental conditions in both tissues analyzed. MTLP could not be considered as a suitable indicator for lead exposure in hepatopancreas.

## 1. Introduction

Penaeid prawns are important subjects of mariculture and coastal fisheries activities throughout the tropics and subtropics [1–3]. Traditionally, prawn farms located near the coast used directly coastal seawater to rear the prawns without additional processes. Shrimp culture is therefore potentially affected by anthropogenic activities including metal contamination. Specifically in coastal Mexican waters, a great number of basic metal industries represent the mainly heavy metal (cadmium, lead, nickel, chromium, etc.) source [4–9]. Penaeid prawns have revealed great capacity to accumulate and take up metals from solution, either essential or not essential elements and yet survive in these polluted environments [10–14]. Consequently, metals are subsequently potentially transferred to man through the food chain. Thus, it is of great concern to investigate detoxificatory processes involved in prawns exposed to metals. It is well known that the induction of metallothionein which is a cystein-rich protein and has detoxifying properties, occurs in aquatic invertebrates after *in situ* or laboratory metal exposure (for review see Amiard et al. [15] and literature cited therein). Moreover, physiological markers reflecting the energetics of an organism may contribute to the understanding of the mode of the toxicant [16–18]. The “Metabolic cost” hypothesis suggests that toxic stress induces metabolic changes which may lead to a depletion of its energy reserves resulting in adverse effects on growth and reproduction [19]. Comprehension of the mechanisms related to the sublethal effects caused by toxic metals upon shrimp metabolism would help to develop sensitive and diagnostic tools (biomarkers) with a predictable capacity in assessing the toxic effects, thus contributing to better coastal seawater management and sustainable aquaculture.

This study is part of a wider investigation of the regulation and accumulation of the trace metals nickel and lead in a penaeid prawn *Penaeus vannamei *[20]; metals previously detected in coastal Mexican waters [4–8]. Nickel has occasionally been interpreted as an essential metal [21, 22], probably as a consequence of its ability to be substituted for other divalent metal ions, particularly zinc, or as a part of an enzymatic structure [23, 24].

This paper specifically investigates both metallothionein-like proteins and energy reserve levels in the hepatopancreas and abdominal muscle of *Penaeus vannamei*, after sublethal exposure of the prawns to dissolved nickel and lead.

## 2. Material and Methods

### 2.1. Experimental Design

Juveniles of* Penaeus vannamei* (between 1–3.0 cm total length; mean dry weight 11.0 ± 8.0 mg; [25]) were obtained from cultures at Aquapacific S.A de C.V., Mazatlan, Sinaloa, Mexico. No distinction between genders was as yet discernible.

Prawns were maintained in artificial sea water (Instant Ocean (IO), Aquarium Systems) at 25 salinity (pH 7.8), 12 : 12 light/dark periods and 25°C as previously described [13, 14]. Animals were fed every two days with commercial flakes (Tetramin) for 15 minutes maximum, before water changes. The use of artificial seawater in all experiments provided physicochemical stability and replicability for trace metal uptake tests [26].

The dissolved metal concentrations used in laboratory exposure of prawns are environmentally realistic for contaminated Mexican coasts [27, 28].

The experimental design involved nine separate experimental groups containing ten prawns each: controls (not metal exposed; three groups), prawns exposed to 55.5 *μ*g L^−1^ nickel (two experimental groups), 191 *μ*g L^−1^ lead (two experimental groups), and same concentration of nickel/lead mixture (two experimental groups) in IO at 25 salinity and 25°C. The hepatopancreas and abdominal muscle were removed from one control group at the beginning of the experiment, as described by Nunez-Nogueira and Rainbow [13]. Prawns from the remaining two control and metal-treated groups were dissected after 10 days of exposure.

## 3. Metal and Metallothionein-Like Proteins Analyses

Samples destined for biochemical analysis were treated as described by Nunez-Nogueira et al. [29]. Briefly, samples were transferred into plastic vials to be immediately frozen in liquid nitrogen for a few minutes. After freezing, samples were freeze-dried overnight and their dry weight measured. Then, samples were homogenized in a buffer solution (20 mM TRIS, 10^−5^ mM *β*-mercaptoethanol, 150 mM NaCl solution adjusted to pH 8.6). The soluble and insoluble fractions were separated by centrifugation at 25,074 g for 55 minutes at 4°C (Biofuge 28 RS, Heraeus Sepatech). The cytosolic heat-stable compounds including metallothionein were isolated by centrifugation of the soluble fraction (12000 g for 10 minutes at 4°C) after heat-treatment (75°C for 15 minutes). In the heat-denaturated cytosol, the amount of MT was determined by differential pulse polarography (DPP) according to Thompson and Cosson [30] and Olafson and Olsson [31]. A MDE 150 Stand Polarographic (Radiometer Copenhagen) Tracelab 50, controlled by the computer software Tracemaster 5 through a Polarographic analyser POL 150 was used. The temperature of the cell was maintained at 4°C. The method of standard addition was used for calibration with rabbit liver MT (SIGMA Chemical Co., St Louis, MO, lot. 20k7000; M-7641 code) in absence of a shrimp MT standard. Polarographic determination in heat-denaturated cytosol is an analytical procedure based on several characteristics of MTs, but it does not allow, with certainty, the assertion that the target molecule is a true MT unless purification and sequencing are carried out. Strictly, therefore, what has been measured is the concentration of proteins with metallothionein properties, that is, metallothionein-like proteins (MTLP).

Metal analysis was carried out in the soluble and insoluble fractions. Nalgene bottles were used to store all reagents. All glass labware was soaked in 10% HCl, rinsed three times with deionized water and dried in a desiccator protected from atmospheric dust. The insoluble and soluble fractions were heated (75°C, 12 h) with suprapure HNO_3_ acid (Carlo Erba). After digestion, metal levels in these acid solutions were determined after dilution with deionized water by flame AAS using the Zeeman effect (Shimatzu 8600-AA spectrophotometer). The analytical method has been described previously by Amiard et al. [32].

Standard addition analyses were performed in an isomedium and concentrations of each element were 25 mg metal mL^−1^ for AAS. The analytical methods were validated by external intercalibrations as previously described Nunez-Nogueira et al. [29] and use of reference material Tort-2 (Lobster hepatopancreas) with more than 90% recovery. Total metal concentrations were recalculated from summation of quantities of trace elements in soluble and insoluble fractions determined previously, by combine means procedure [33]. The results were expressed in *μ*g·g^−1^ dry weight of the organ for Ni and ng·g^−1^ in case of Pb, respectively.

## 4. Energy Reserves Analyses

Hepatopancreas and abdominal muscle samples were homogenized with a porcelain mortar and pestle at −2°C. The powder obtained was homogenized in a motorized grinder with 1.5 mL citrate buffer (pH 5.0) for glycogen and lipid analyses. Total lipids were determined by the sulfophosphovanillin reaction, according to Frings et al. [34], while glycogen concentration were determined in two aliquots of the homogenate, using enzymatic digestion by amyloglucosidase, according to Carr and Neff [35]. Olive oil and glycogen from oysters (Sigma type III) were used as standards for each method, respectively.

### 4.1. Statistical Analysis

Students* t-*test (*P* < .05) were performed for protein, lipids, glycogen, and metal concentration comparisons. Tests were developed for small samples according to Williams [33], and carried out in STATISTICA 5.1 for windows (StatSoft Inc.). 

## 5. Results

### 5.1. Metallothionein-Like Protein Concentrations

The MTLP concentrations in hepatopancreas and abdominal muscle of juveniles of *Penaeus vannamei* are illustrated in Table 1. MTLP concentrations were higher in hepatopancreas than in abdominal muscle in all experimental organisms (control, Ni exposed, Pb exposed, Ni+Pb exposed). In hepatopancreas, the difference in MTLPs concentrations between the control and Ni-treated shrimps were not significant, while a significant increase in MTLP concentrations was observed in Pb-treated and metal mixture-treated organisms *versus* controls. The MTLP concentration ranged from 6.83 to 31.04 mg g^−1^ in controls, 9.46 to 62.04 in Ni-treated, 31.79 to 121.85 in Pb-treated, and 36.11 to 94.83 mg g^−1^ in metal mixture-treated, respectively. In abdominal muscle, a significant increase in MTLP concentrations was observed in Ni-treated and Pb-treated prawns in comparison with controls whereas no differences in MTLP concentrations were detected in metal mixture treated organisms *versus* controls (apparently due to the presence of two muscle samples (out of ten) with higher MTLP concentrations within the Pb-treated prawns, suggesting noninfluence of Ni and/or Pb in MTLP induction in muscle, even when an apparent difference was detected in one metal treated groups). The MTLP concentration in muscle tissue ranged from 0.30 to 0.83 in controls, 0.30 to 0.99 in Ni-treated, 0.54 to 1.54 in Pb-treated, and 0.51 to 0.60 mg g^−1^ in metal mixture-treated, respectively.

### 5.2. Metal Concentrations

**Figure 1:**
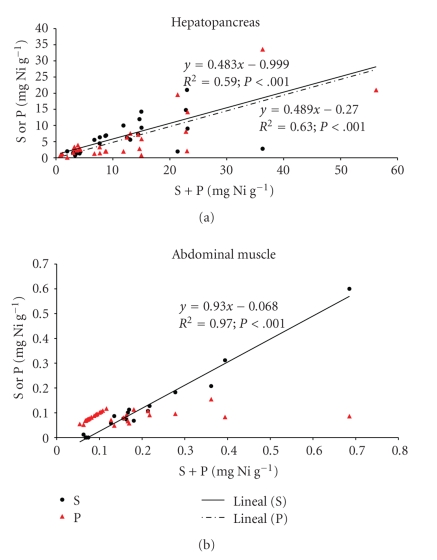
Relationship between soluble (S) or insoluble (P) fraction against total Ni (S + P) in the hepatopancreas and abdominal muscle of juveniles of *Penaeus vannamei* (*n* = 31) after 10 days of exposure to 55.5 mg Ni L^−1^ and 191 mg Pb L^−1^ or both in a mixture (MM) at 25°C and 15 ups, respectively.

Ni and Pb concentrations (total, soluble, insoluble) in the hepatopancreas and abdominal muscle of *Penaeus vannamei *are indicated in Tables 2 and 3, respectively. Ni concentrations were significantly higher (*P* < .05) in the hepatopancreas *versus* the abdominal muscle of prawns, Pb was found in major quantities in abdominal muscle. The measured total Ni concentration in hepatopancreas ranged from 3.67 to 21.37 *μ*g g^−1^ (controls), 1.98 to 11.86 (Ni-treated), 1.11 to 22.84 (Pb-treated), and 3.86 to 56.23 *μ*g g^−1^ (mixture-treated), respectively. Due to a great individual variability, no significant differences were observed among groups, including Ni-treated. In abdominal muscle, Ni concentration ranged from 0.02 to 0.03 *μ*g g^−1^ in each group and not differences against controls were observed (Table 2). The distribution of Ni among the soluble and insoluble fractions in the hepatopancreas and the abdominal muscle of prawns are shown in Figures 1(a) and 1(b), respectively. In hepatopancreas, this metal was nearly equally distributed between soluble and insoluble fractions (Figure 1(a)) whereas in the abdominal muscle (Figure 1(b)), the soluble fraction was more important than the insoluble fraction.

Lead was detected only in the insoluble fraction of both tissues (Table 3). The mean Pb concentrations in all groups were below 0.17 mg Kg^−1^ in abdominal muscle and 0.003 mg Kg^−1^ in hepatopancreas. A slight increase (not significant) was observed in the hepatopancreas in prawns exposed to this metal, either alone or in a mixture.

### 5.3. Energy Reserves Analyses

**Figure 2:**
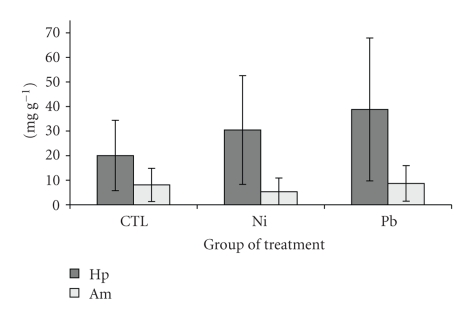
Lipid concentration (mg g^−1^; mean ± *sd* ) in hepatopancreas (Hp) and abdominal muscle (Am) of *Penaeus vannamei* after 10 days of exposure.

**Figure 3:**
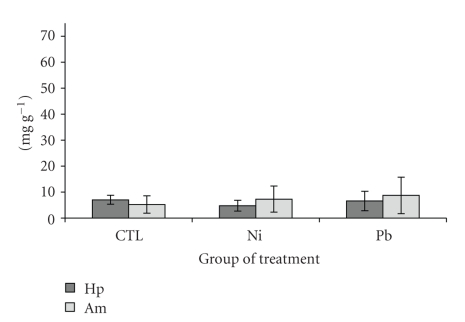
Glycogen concentration (mg g^−1^; mean ± *sd* ) in hepatopancreas (Hp) and abdominal muscle (Am) of *Penaeus vannamei* after 10 days of treatment. *Significantly different from control (*P* < .05).

The concentration of lipids and glycogen, determined in hepatopancreas and abdominal muscle are shown in Figures 2 and 3, respectively. Lipid concentration in abdominal muscle ranged from 1.08 to 16.45 mg g^−1^ dw (controls) and from 0.86 to 18.30 mg g^−1^ (metal-treated). In hepatopancreas, lipid concentration ranged from 1.85 to 48.95 mg g^−1^ dw (controls) and from 6.53 to 88.66 mg g^−1^ (metal-treated). No significant differences were observed between controls and treated prawns in the case of lipids. The range of glycogen was from 0.51 to 23.60 mg g^−1^ (controls) and 1.60 to 15.05 mg g^−1^ (metal-treated) in abdominal muscle, while it was from 4.72 to 8.89 mg g^−1^ (controls) and 1.07 to 11.33 mg g^−1^ (metal-treated) in hepatopancreas, respectively. Glycogen concentration in abdominal muscle from Pb-treated prawns was the only one significantly (*P* < .05) different from controls. In this case, muscle showed an increase in glycogen.

## 6. Discussion

Different studies have shown the presence of heavy metals, including Ni and Pb in Mexican coastal waters [4–6]. For example, coastal zones in Tabasco and Veracruz States (Gulf of Mexico) have shown lead concentration in water between 65 to 210 *μ*g L^−1^, while others like Cr, Hg, and Cd between 0.5 and 15 *μ*g L^−1^ [27]. These concentrations can be above the national recommended level (local legislation), as have been observed in the Mexican Pacific coast [28]. The Pacific white prawn *Penaeus vannamei* spends part of its life cycle in coastal or estuarine areas, making it suitable for metal exposure. This species showed capacity to induce metallothioneins by metal exposure [36, 37] and accumulate metals [12, 25]. Total Ni concentrations found here were in good agreement with previous studies that show Ni regulation in decapod crustaceans [38, 39], including *P. vannamei* [20]. Nickel was found almost equally distributed between the soluble and insoluble fraction (Table 2). These concentrations are within values previously described in Penaeid prawns (including *P. vannamei*) [40, 41] for these tissues. There was no significant increase in total Pb concentration as well. The reason of this lack of increase compared to controls must be related to the fact that both metals are regulated or partially regulated (in case of lead as a nonessential metal) by this prawn. Previous studies have shown that Ni can be regulated by decapods crustacean [39, 42] providing a capacity to maintain a minimal trace level concentration in their bodies. In case of Pb, Vogt, and Quinitio [43] suggested that this non-essential metal is eliminated by forming insoluble-lead rich deposits that are excreted after lysosome autolysis, process that can include MT content during protein turnover [44] (see below). Due to that metal exposures do not exceed the LC-50 for 96 hours in both cases for the experimental conditions here tested, and are far below these values (60.54 mg L^−1^ for Ni and 6.22 mg L^−1^ for Pb, resp.), suggests that such a trace level of exposures is not enough to reach the threshold level of regulation in case of *Penaeus vannamei* without significant accumulation. 

According to the results obtained here, MTLP appear to be induced by Pb in hepatopancreas under the experimental conditions tested (Table 1). Prawns exposed only to Ni in solution did not show a significant increase in tissue concentration, while prawns treated with Pb either alone or in a mixture with Ni, almost double the amount of MTLPs observed in controls and Ni-treated groups (Table 1).

Most of the lead was detected in the insoluble fraction of the hepatopancreatic samples (Table 1), suggesting a nonsoluble lead-rich deposit. Some authors have suggested that MT's induction is an intermediate step before insoluble deposits are formed [45, 46]. Lead-insoluble deposits or granules have been observed in terrestrial and aquatic invertebrates [47–51]. Rainbow [52] highlighted that isopods accumulated metals in detoxified granules (non-soluble) with rapid elimination of lead, compared to other metals (e.g., cadmium). Complementary to Vogt and Quinitio [43], our results suggest that lead is mainly stored and detoxified within granules, instead to MTLP's, perhaps at the antennary gland as in* Penaeus monodon*, and excreted as an apocrine secretion in the urine [49, 50], not only at high-lead level of exposure, as was proved in *P. monodon*. In this way, lead appears to be treated as some essential metals, in respect of quelating cytosolic molecules. This difference in detoxification between essential and non-essential metals was already observed in *P. vannamei *[37] and *Penaeus indicus*, where MTLPs appear to be the first detoxification strategy involved in non-essential metals exposure, while essential metals are regulated in cytoplasm by another method (e.g., insoluble deposits [29]). It is also considered that MTLPs increase slightly with time and doses as a result of a constant turned over [53]. In the abdominal muscle, slight increase in MTLP concentration was observed in Ni- and Pb-treated, but not in a mixture (Table 1). No significant relationship (not shown) between soluble Ni and MTLP was observed. Comparing the total metal concentration between controls and treated groups in muscle, no significant increase was detected, suggesting that these increases in MTLP could be related to other factors, like normal homeostasis of other essential metals (e.g., Zn or Cu) and inflammation process caused during experiment procedure, rather than metal exposure [15, 54, 55]. This idea is also supported by the lack of MTLP induction in muscle in metal mixture-treated group (Table 1). 

It has been proved, that longer period of exposure and higher metal concentrations induced MTLPs in crustaceans and aquatic or terrestrial invertebrates [36, 56–58]. Our results obtained here suggest that it is necessary to carry out bioassays with different metal concentrations near (below) the LC50, to identify the threshold level for MTLP induction for these metals in this species. A recent work in our lab, revealed that using the following metal concentrations: 15.6, 31.3, 62.5, 125 and 250 mg L^−1^ for Ni and 4.68, 5.88, and 7.41 mg L^−1^ for Pb, for 96 hours of exposure, are higher than the environmentally realistic metal concentrations tested (55.5 *μ*g L^−1^ for Ni and 191.2 *μ*g L^−1^ for Pb, resp.), and far below the lethal doses obtained, in good agreement with our results, where the trace level of exposure only inducing minimal or no detectable changes in the metal body concentrations. 

It is widely recognized that both Ni and Pb share chemical properties and features with class B and Borderline metals, like copper, zinc, arsenic, cadmium, and mercury [59, 60]. Within these metals, cadmium, copper, mercury, zinc, and arsenic have been identified as metallothionein inductors [56, 57, 61–64]. Due to these, two possibilities emerge for MTLP induction by Ni and Pb. The first involves zinc/copper substitution by these metals in the active sites of the cystein-rich proteins (metal-clusters) or secondly by direct generation of metal element responses (MERs) inside the cells. In the first case, nickel or lead compete against zinc for the active clusters sites of the MT or generate an exchange Ni/Pb versus Zn, inducing and increase in the zinc intracellular concentration and its bioavailability, promoting an homeostatic response by increasing the amount of MTs. This phenomenon has been observed in other invertebrates [56, 63] and vertebrates [65, 66], and metal substitution in metallothioneins has been registered in different cases [56, 58, 64], for example, cadmium in crustaceans [57]. In the case of MERs production, the MTLP induction might be related to free-radicals generation and/or oxidative damage that activate a metal transcription factor capable to interact with the promotor region of the MT gene by a positive feedback [64]. These results are in good agreement with previous nickel and lead reports as MT inductors, supporting that this inductive capacity is present not only in *in vitro* tests [63, 66].

One interesting observation relay on the relationship observed between the amount of MTLPs concentration in hepatopancreas of controls and the whole body weight, which almost reaches 3% (Table 3). Metallothioneins are considered as homeostatic proteins, widely present in aquatic and terrestrial invertebrates either from contaminated and noncontaminated sites [56, 61, 67–69]. An explanation could be related to a reduction in the tissue weight, due to lower quality food used during captivity, or stressful conditions during acclimation, producing a minor difference between tissue mass and MTLP content. Weight is a factor proved that affect the MT concentration in soft tissues of aquatic invertebrates [67, 68], however, this appears to be scarcely possible due to the fact that mean weight of the hepatopancreas was between 8 and 9 mg (dry weight) in all experimental groups, including controls, representing nearly 6% of the whole body weight, in good agreement to the proportion established in healthy decapods [70]. Another more suitable explanation depends on the previous exposure to the metals. The hepatopancreas of *P. vannamei* showed traces of Pb and relatively amounts of Ni in controls (0.002 and 6.41 *μ*g g^−1^, resp.), which might be triggering the defensive and regulatory mechanisms of MTs, explaining why the MTLP concentration in this group almost reaches 3% of the tissue weight, highlighting that previous metal exposures increase the original amount of MTLPs [61].

Higher concentrations of energy reserves, mainly lipids, and MTLPs in the hepatopancreas are in good agreement with its function of main organ for metabolic reserves and metal-binding proteins [71]. Energy reserves are of importance considering (among others) its use in detoxification processes [72–74]. In this respect, the concentration of lipids (Figure 2) and glycogen (Figure 3) did not change in the hepatopancreas. In abdominal muscle, a slight increase in the lipid and glycogen fraction occurred, suggesting a possible effect of Pb on these reserves, but no correlation was observed with metal concentration in the tissue. Toxic Pb exposures have been considered a cause for a decrease in metabolic rates in postlarvaes of *P. indicus* (according to Satyavathi [75] and Chinni and Yallapragada [73]), causing perhaps the higher glycogen concentration observed in the abdominal muscle, as a result of lower degradation rates by glycogen phosphorylase and an increase in the carbohydrate synthesis by the glycogen synthase [76]. However, the low Pb concentration detected in the muscle, suggested that other stressful factors could be involved, and not necessarily related to the metal presence in the tissue. Previous studies in prawns and other crustaceans, have shown that under some particular stressful conditions (e.g., salinity and metal conditions [55]), the energy budget can be modified in opposite direction. In some cases, energy production can be altered by metals like mercury and zinc by increasing the lactate dehydrogenase activity, enzyme involved in energy production, as was observed in the crab *Carcinus maenas* [77]. The fact that no significant changes were observed in the hepatopancreas, which showed to be the main target organ for Pb, suggests that the concentration of metal in solution tested in this study is not sufficient to compromise the amount of energy available in these tissues, and it is enough to cover the detoxification process trigger under the stress simulated in the hepatopancreas [74], or involves alternative mechanisms to mitigate the adverse effect [73]. It is possible that higher metal concentrations, near lethal concentrations and longer exposure periods, could induce significant changes in the energy reserve, as has been observed in *P. indicus *exposed to Pb [73], but further studies are required in *P. vannamei*.

## 7. Conclusions

This study shows the first results of MTLP quantification and energy reserves in the estuarine prawn *P. vannamei* exposed in the laboratory to Ni and Pb. It is assumed that the MTLP induction in the hepatopancreas is caused by metal stress (presence of Pb and Ni) and did not influence the energy metabolism. This protein induction appears to be related either to the detoxification process of this non-essential metal (Pb) and by essential metal regulation (Ni). In case of Ni, the MTLP concentration could be considered as the basal level for cellular homeostasis of the essential metal. The experimental conditions tested do not modify the energy budget present in both tissues at the sublethal exposure tested. MTLP concentrations could not be considered as a biomarker, at least in the hepatopancreas of *P. vannamei*, for the realistic metal concentration tested here, in environmental risk studies. Further studies involving different metal concentrations (just below the lethal level) and exposure times will help to define the threshold level for MTLPs and energy reserves changes in this species.

## Figures and Tables

**Table 1:** MTLP concentration and energy reserves (mean ± standard deviation) in control and metal-treated in abdominal muscle and hepatopancreas from *P. vannamei*.

Group	MTLP (mg g^−1^)		Energy reserves (mg g^−1^)			
	Abdominal Muscle (*n* = 10)	Hepatopancreas (*n* = 10)	Abdominal Muscle (*n* = 6)		Hepatopancreas (*n* = 6)	
			Lipids	Glycogen	Lipids	Glycogen

Control	0.51 ± 0.17	29.44 ± 16.49	8.10 ± 6.77	5.22 ± 3.34	20.07 ± 14.33	7.04 ± 1.74
Ni-treated	0.74 ± 0.22*	37.09 ± 15.36	5.36 ± 5.56	7.29 ± 5.04	30.45 ± 22.14	4.77 ± 2.05
Pb-treated	0.93 ± 0.51*	61.70 ± 34.59*	8.05 ± 7.26	9.67 ± 1.34*	38.79 ± 29.10	6.57 ± 3.74
Mixture-treated	0.56 ± 0.04	62.36 ± 20.06*	—	—	—	—

*Significantly different from respective control (Student *t*-test; *P* < .05).

**Table 2:** Nickel concentration (*μ*g g^−1^) in abdominal muscle and hepatopancreas from *P. vannamei, *at the soluble and insoluble fraction.

Tissue	Group	Total		Soluble		Insoluble	
		Mean	SD	Mean	SD	Mean	SD
Abdominal muscle	Control	0.30	0.19	0.23	0.18	0.10	0.02
	Ni-treated	0.13	0.04	0.07	0.04	0.08	0.03
	Pb-treated	0.08	0.01	N. D.*	N. D.	0.08	0.02
	Mixture-treated	0.08	0.01	N. D.	N. D.	0.08	0.01

Hepatopancreas	Control	6.41	3.65	2.98	2.27	3.43	2.86
	Ni-treated	6.12	3.47	4.17	3.28	1.95	1.18
	Pb-treated	8.82	6.84	7.32	5.10	2.97	2.56
	Mixture-treated	23.09	16.44	13.65	11.10	11.14	11.12

*Not detected.

**Table 3:** Lead concentration (mg Kg^−1^) in abdominal muscle and hepatopancreas from *P. vannamei, *according to the soluble and insoluble fraction.

Tissue	Group	Soluble		Insoluble	
		Mean	SD	Mean	SD
Abdominal muscle	Control	N. D.	N. D.	0.16	0.03
	Ni-treated	N. D.	N. D.	0.17	0.07
	Pb-treated	N. D.	N. D.	0.14	0.06
	Mixture-treated	N. D.	N. D.	0.15	0.04

Hepatopancreas	Control	N. D.	N. D.	0.002	0.001
	Ni-treated	N. D.	N. D.	0.005	0.002
	Pb-treated	N. D.	N. D.	0.008	0.007
	Mixture-treated	N. D.	N. D.	0.014	0.009

*Not detected.
